# XMRV and Public Health: The Retroviral Genome Is Not a Suitable Template for Diagnostic PCR, and Its Association with Myalgic Encephalomyelitis/Chronic Fatigue Syndrome Appears Unreliable

**DOI:** 10.3389/fpubh.2017.00108

**Published:** 2017-05-22

**Authors:** Simona Panelli, Lorenzo Lorusso, Alessandro Balestrieri, Giuseppe Lupo, Enrica Capelli

**Affiliations:** ^1^Department of Earth and Environmental Sciences, Section of Animal Biology, University of Pavia, Pavia, Italy; ^2^Centre for Health Technologies (C.H.T.), University of Pavia, Pavia, Italy; ^3^Neurology Unit, A.S.S.T. Franciacorta, Chiari (Brescia), Italy; ^4^Department of Biosciences, University of Milano, Milano, Italy

**Keywords:** ME/CFS, retroviruses, XMRV, murine leukemia virus, PCR, retroelements, ERV, mouse

## Abstract

A few years ago, a highly significant association between the xenotropic murine leukemia virus-related virus (XMRV) and myalgic encephalomyelitis/chronic fatigue syndrome (ME/CFS), a complex debilitating disease of poorly understood etiology and no definite treatment, was reported in *Science*, raising concern for public welfare. Successively, the failure to reproduce these findings, and the suspect that the diagnostic PCR was vitiated by laboratory contaminations, led to the retraction of the paper. Notwithstanding, XMRV continued to be the subject of researches and public debates. Occasional positivity in humans was also detected recently, even if the data always appeared elusive and non-reproducible. In this study, we discuss the current status of this controversial association and propose that a major role in the unreliability of the results was played by the XMRV genomic composition in itself. In this regard, we present bioinformatic analyses that show: (i) aspecific, spurious annealings of the available primers in multiple homologous sites of the human genome; (ii) strict homologies between whole XMRV genome and interspersed repetitive elements widespread in mammalian genomes. To further detail this scenario, we screen several human and mammalian samples by using both published and newly designed primers. The experimental data confirm that available primers are far from being selective and specific. In conclusion, the occurrence of highly conserved, repeated DNA sequences in the XMRV genome deeply undermines the reliability of diagnostic PCRs by leading to artifactual and spurious amplifications. Together with all the other evidences, this makes the association between the XMRV retrovirus and CFS totally unreliable.

## Introduction

Myalgic encephalomyelitis/chronic fatigue syndrome (ME/CFS) is a complex, debilitating disease of poorly understood etiology. It follows a prolonged course, with relapses and remissions, and is characterized by persistent, unexplained fatigue associated with impaired memory or cognition, pain, and a diversity of immune, neurological, and autonomic symptoms (1). A cardinal feature is the worsening of symptoms following minimal physical or mental exertion that can persist for days or weeks and is not relieved by sleep. There are therapies that help patients to minimize some symptoms, but no definitive treatment is available. The illness is globally endemic, with a prevalence estimated around 0.11–0.19% of adult population in the UK (2) and 0.4% in the USA, where it affects over 800,000 adults (3). Annual direct costs for medical care are estimated as 7 billion dollars (3); considering also indirect costs in terms of, e.g., lost productivity and social costs for patients and families, it becomes clear that ME/CFS represents a serious challenge for clinical medicine and public health.

Usually, ME/CFS cases are sporadic, but occasional outbreaks have been reported, with geographic and/or temporal clusters [see, for example, Ref. (4, 5)]. A widely studied outbreak of ME/CFS took place in the lake Tahoe region (Northern Nevada/California) from 1984 to 1987 and was recorded in 259 patients (6).

A dysfunction in the body’s response to infection is known to play a major role in the onset of ME/CFS (7), even if etiology and underlying mechanisms are poorly understood. Signs of autoimmunity and metabolic disturbances are often found (8). Infectious origins have been invoked as prodromal events: proposed viral candidates include Epstein–Barr virus, human herpesvirus 6, Borna disease virus, and enteroviruses (9–11). Other proposed agents include bacteria (*Borrelia burgdorferi* and *Coxiella burnetii* among others) and fungi (*Candida albicans*) (10).

In 2009, *Science* published a paper by Lombardi et al. that established a causal relationship between ME/CFS and a murine xenotropic retrovirus (i.e., integrated into the animal’s genome, but not able to infect cells from that species): the xenotropic murine leukemia virus-related virus (XMRV). XMRV sequences were isolated by a diagnostic PCR in 67% of ME/CFS patients vs. 3.7% of healthy controls, and authors claimed the detection of anti-XMRV antibodies in the sera of ill patients as well as isolation of infectious virus from patients’ CD4+ T cells.

Incidentally, XMRV was the same virus linked, a few years before, by Urisman and co-workers, to prostate cancer (PC) in patients with impaired innate antiviral activity caused by a mutation in Rnase L gene (12).

The Lombardi et al.’s paper was considered a major scientific breakthrough and provoked a remarkable echo in the public opinion and concern for public welfare. Indeed, the link between XMRV and either PC or CFS/ME raised the fear for a new HIV-1 case: a retroviral pathogen potentially able to be transmitted between individuals through blood supplies from healthy donors [as reported in Ref. (13)] and that had spilled over from mice to humans (14).

Unfortunately, in the case of ME/CFS, before this claim had been fully validated, many patients embraced XMRV as the long-sought causal agent and began considering potential treatments. Because of the similarities between the mouse virus and HIV, some of them even started taking AIDS drugs (15).

An eruption of studies rapidly followed, which investigated independent patient cohorts for XMRV. Most of these papers failed to find any association or causal relationship at all, with both PC and CFS/ME [e.g., Ref. (16–19)]. Among these, a multi-blinded trial, co-authored by the original investigators who described the association, revealed no evidence at all of either XMRV or other murine leukemia virus (MLV) infection in a cohort of rigorously characterized, geographically diverse populations of ME/CFS patients (10). However, the public alarm raised by these results was such that, despite these clear inconsistencies, FDA voted in favor of an indefinite deferral to donate blood of all individuals with a medical history/diagnosis of ME/CFS (20).

Overwhelming evidence was then produced that XMRV is a laboratory-derived virus, with uncertain diffusion in the “real world,” which originated in the mouse genome from the recombination between two endogenous proviruses (PreXMRV-1 and PreXMRV-2) during the process of production of the PC *22Rv1* cell line, in particular, when passing the primary tumor tissue in nude mice [Ref. (14) for a review]. In this regard, Das Gupta and co-workers clearly demonstrated the absence of XMRV and other related viruses in the primary PC tissue used to generate the *22Rv1* cells, in terms of either PCR assays, *in situ* hybridizations, infectious XMRV, or the presence of antibodies against the virus (21).

New data were also collected showing that the amplification of XMRV sequences from patients had originated from poor laboratory practices, particularly from contaminations due to the widely used *22Rv1* cell line and to the presence of mouse DNA in widely used laboratory reagents: DNA extraction columns, *Taq* polymerases (particularly the ones containing mouse monoclonal antibodies, MAbs) and RT-PCR enzymes (22) but also other preparations containing MAbs, as antibodies to CD4, CD8, and CD14 (23).

Finally, virological assays were attempted on ME/CFS blood samples previously identified as XMRV positive for detecting the presence of: (i) infectious XMRV or MLV particles; (ii) XMRV-specific antibodies (23). No evidence of XMRV or MLV was found in these samples by any of the multiple methodologies used. In addition, sera from CFS and healthy controls, most likely through the complement fraction, were found to inhibit these viruses, raising serious concerns about the possibility of a successful XMRV infection in humans.

The inevitable consequence was the full editorial retraction by *Science* of the paper by Lombardi et al. (24). Other papers that had played crucial roles in this story were retracted: (i) the report by Urisman et al. (12) on the association with XMRV-PC (25); (ii) the paper by Lo et al. (13) which had detected XMRV in blood donors, proved to be vitiated by mouse DNA contamination (26).

Since then, XMRV remained an elusive entity and the latest case in the list of “human retroviruses” with a proposed chronic disease or tumor association that did not pan out (27). Nevertheless, it continued to be the subject of researches and debates. As an example, it has been recently included among the zoonoses screened in a survey of forestry workers, with a seropositivity of 0/722 analyzed samples (28). Some PCR positivity in human patients has been still reported (29, 30). Once again, results suffer from poor reproducibility, e.g., when using independent genomic DNA preparations from the same individuals, and have been justified as due to contaminations of PCR reagents with murine DNA (14, 30) or to air-driven amplicon contaminants (29).

However, as already underlined by some researchers, contamination alone does not seem to adequately explain the detection of XMRV by Lombardi et al., especially for what concerns the diagnostic PCR (23).

With the current paper, we would like to suggest that at the origin of these misinterpretations and elusive PCR data, which generated frustration in many patients with regard to the possible availability of therapies for diseases with no treatment, there could have been other co-causes that played major roles, in particular:

(i) Aspecific, spurious annealings of the primers described by Lombardi et al. in a number of sites of the human genome.
(ii) Strict homologies between the PCR target (XMRV) and interspersed repetitive elements (in particular, retroelements) widespread in mammalian genomes that would make any diagnostic PCR totally unreliable.

## Another Piece in the Puzzle?

To verify whether the above-mentioned points have the potential of playing substantial roles in explaining this story, we planned a proof-of-concept survey of DNA sequence databases, and namely:

(i) A bioinformatic analysis on the primer sequences described by Lombardi et al. (31), compared against human and murine (house mouse *Mus musculus*) genomes.
(ii) A broader bioinformatic analysis searching the same genomes for homologies of the whole XMRV (NCBI Acc. No. EF185282) and parental PreXMRV-1 (FR871849.1) and PreXMRV2 (FR871850.1) sequences.

## Bioinformatic Analysis on the Primers by Lombardi et al.

The first pair of primers used for the XMRV diagnostic PCR targets the viral GAG gene (F 5′-ATCAGCTAACCTACCCGAGTCGGA-3′; R 5′-GCCGCCTCTTCTTCATTGTTCTC-3′) with an expected molecular weight of 736 bp. The second pair anneals to the ENV gene (F 5′-GCTAATGCTACCTCCCTCCTGG-3′; R 5′-GGAGCCCACTGAGGAATCAAAACAGG-3′) and produces a 352-bp amplicon. Both pairs are used to screen DNA from peripheral blood mononucleated cells (PBMCs) of ME/CFS patients and healthy controls, with a low stringency PCR program including 45 amplification cycles.

We performed similarity searches for both pairs by means of the basic alignment search tool program within the GenBank database,^1^ using the available specialized murine and human genomic pages and their default parameters. The following criteria were used to analyze the results: (i) only the first 50 BLAST results were considered; (ii) among them, only the hits encompassing at least the final 12 nucleotides at the 3′ end of the primer sequences were selected. In addition, homologies for reverse (R) primers were searched only in the BLAST entry that produced the best expected value (E-value) for the corresponding forward (F) primer companion. Based on the results (Table 1), both pairs of primers appear highly susceptible to produce non-specific amplifications, especially the ENV pair. In addition, due to the stringent criteria used for our analysis, the number of possible mismatches is likely to be underestimated, especially in the context of a 45-cycle PCR, which is *per se* likely to produce a large number of artifacts. The presence of multiple primer targets may lead, e.g., to the formation of chimeric DNA molecules; PCR competition among the various products, for its part, may explain the irreproducible results and extra-bands which have been often reported in XMRV studies. Finally, the same oligonucleotide, in these PCR conditions, may act simultaneously as F and R primer (32).

**Table 1:** **Bioinformatic analyses on XMRV**.

Primer	Length (ntt)	Number of hits		Accession no. showing the best *E*-value	
		
		*Homo sapiens*	*Mus musculus*	*H. sapiens*	*M. musculus*
ENVF	22	17	18	NC_018927.2 (chr. 16)	AC_00027.1 (chr. 5)
ENVR^a^	26	8	8	–^b^	–^b^
GAGF	24	–^c^	5	–	AC_00030.1 (chr. 8)
GAGR^a^	23	2	2	NC_018914.2 (chr. 3)	–^b^

*Hits produced by searching the primers by Lombardi et al. (31) against the BLAST human and mouse genome pages. Only the first 50 BLAST results were considered and, among them, hits encompassing at least the final 12 nucleotides at the 3′ end of the primer sequences were selected*.

*^a^The sequence was reverse complemented before searching*.

*^b^The reverse primers were searched against the same Acc no. producing the best E-value in their forward companions*.

*^c^Best results were three 100% identities of nucleotides 10–23*.

## Bioinformatic Analysis of the Whole Genome of XMR Virus and Parental Proviruses

All the three retroviruses showed homologies on the near totality (>90%, typically 97–100%) of their length (about 8 kbp) with multiple regions of the murine genome. Table 2 shows the detailed results for XMRV, while those for PreXMRV-1 and PreXMRV-2, being very similar, have not been reported for the sake of clearness.

**Table 2:** **Bioinformatic analyses on XMRV**.

Genome	Acc no. producing hits with XMRV	%homology	%coverage	Region of the hit producing the homology	Annotation of the region
Mouse	NC_00079.6 (chr. 13 of strain C57BL/6J)	93	99	Multiple	ERV-K; various uncharacterized protein-coding loci
	AC167466.6 (chr 7, clone RP24-220N8)	94	99	7918-20742	MIR, L1, RTLR4_MM
	AC121813 (chr 9, clone RP23-457E5)	94	99	37845-45763	ERV1
	AC161413.5 (chr 19, clone RP23-106D17)	94	99	30251-38177	RTLR4_MM

Human	AC084198,31 (chr 3, clone RP11454H13)	67	10	39494-40066	HERV-HC2
	NG_001019.6 (chr 14, IgH locus)	68	18	3 matches of a 500-bp unit plus other minor matches	Intergenic region among V segments
	XR_945967	66	12	2888-3497; 4124-4692	Long ncRNA
	AC078899.1 (chr 19, clone CTC-575C13)	68	12	151757-152247; 153125-153501	Located among Alu and LTR6 repeats
	AC073174.5 (chr 10, clone RP11-319F12)	67	12	109485-110094; 1100856-111289	Not annotated

*Examples of hits produced by comparing XMRV (EF185282) genome against the BLAST human and murine genome pages*.

The homologous murine regions are in general annotated as being composed of interspersed repeats and retroelements, often of viral origin, as in the case of NC_000079.6 and, within this entry, of the hit with the endogenous retrovirus group K (ERV-K). Other hits on the same entry include “uncharacterized protein loci” that, if searched against BLASTP, show identity with several retroviral gag-pro-pol polyproteins belonging to XMRV and to a number of other MLV. Identities are always >97%, coverage >90% and *E*-value = 0. Other murine repetitive elements producing significant hits are the widespread mammalian interspersed repeats (MIRs) and LINE 1 (L1) sequences (AC167466.6), the endogenous retrovirus 1 (ERV1) (AC121813), and the RLTR4_MM repeats (AC167466.6 and AC161413.5). This last repeat is part of the long terminal repeat (LTR) family of retroelements, typical of the murine genome, which has been already shown to be highly homologous to MLV.^2^

The human genome, for its part, shows shorter but significant hits, e.g., a long non-coding RNA (ncRNA) (see the Acc no. XR_945967), a human ERV (HERV-HC2, AC084198.31) and the immunoglobulin heavy chain locus (NG_001019.6). Moreover, the XMRV genome results largely composed by blocks retrievable in the human genome as part of (or in close proximity with) interspersed widespread repeated elements, as HERVs, Alus, LTRs. Accordingly, the XMRV sequence “blocks” producing the near totality of human hits (ntt 2,755–3,366; 4,124–4,557; 4,871–5,259 of EF185282) are recognized by the RepeatMasker database^3^ as human ERV-class 1 belonging to the LTR class of interspersed repeats.

## Screening of Selected Human and Animal Populations

Finally, the proof of principle presented in this study was completed with an empirical study. Human and animal populations were screened using the primers designed by Lombardi et al. (31) and two new pairs of primers designed in our laboratory with the aim of reducing as much as possible the aspecific annealings. Also, the new pairs target the GAG (F 5′-GGGTCTCCAAAACGCGGGCA-3′; R 5′-CAGGAGGGAGGTCTGGGGCC-3′) and ENV (F 5′-ACTACGAAGGGGTGGCCGTCC-3′; R 5′-GAACCAGGGCCTGCACTACCG-3′) genes, producing amplicons of, respectively, 586 and 915 bp.

In detail, the populations screened were

- a group of 47 patients with the diagnosis of ME/CFS according to Fukuda’s criteria (33) and 53 negative controls (bone marrow donors).
- 11 laboratory mice (strains BDF and Ts65Dn) and 15 laboratory rats (strains Wistar and brown).
- 11 wild rodents (wood mouse, *Apodemus sylvaticus*).
- 92 wild mammals: 25 badgers (*Meles meles*), 30 martens (*Martes* spp.), 14 red foxes (*Vulpes vulpes*), and 23 hares (*Lepus europaeus*).
- 23 bovines, which, to our knowledge, have never been screened before, but have a tight relationship with man.

Among laboratory mice, the BDF strain was derived from C57L/J (positive for PreXMRV1) × DBA/2J (positive for PreXMRV2) strains (34), while the Ts65Dn strain, which represents the murine model for Down syndrome, is a more complex case, derived from several crosses involving four different mouse strains (of which only C3H/HEJ is known to be PreXMRV2-positive) and a screening over two generations (34, 35). Wild animals were screened as potential zoonotic reservoirs according to Jurke et al. (28).

Results are presented in Table 3. The only case of unambiguous amplification of a correctly sized band, the identity of which was confirmed by sequencing, involved the BDF mouse strain, known carrier of the parental proviruses. Non-reproducible bands, often associated with extra-bands, characterized a negligible sample of human patients (that were by far the most numerous group), the second laboratory mouse strain (Ts65Dn, for which we can hypothesize a contribution of PreXMRV2 from one of the progenitor strains) and wood mice. In the last case, the spurious origin of amplicons was confirmed by sequencing (Table 3). We suggest that this sequence, homologous to conserved regions of plant potyviral proteins (36), may be associated with food. No amplicons were ever obtained from rats, bovines, and wild mammals.

**Table 3:** **Results of the screening for XMRV performed on human and animal sampling populations**.

Sampling population	DNA source	*N*	No. of positive samples	No. of negative samples	Primers	Comments
Human ME/CFS patients	Peripheral blood	47	2	45	1 + 2	Amplicons were not reproducible and often associated with spurious extra-bands
Human controls	Peripheral blood	53	0	53	1 + 2	
Laboratory mouse strain BDF	Peripheral blood	1	1	0	1 + 2	Reproducible band of the correct size. Identity was confirmed by sequencing
Laboratory mouse strain Ts65Dn	Tail tip	10	10	0	1 + 2	Scarce reproducibility of independent amplifications. Extra-bands were often observed
Laboratory white rat (Wistar)	Tail tip	10	0	10	2	
Brown laboratory rat (*Rattus norvegicus)*	Tail tip	5	0	5	2	
Wood mouse (*Apodemus sylvaticus*)	Tail tip	11	?	?	2	Very scarce reproducibility: two samples were positive in 4/7 repetitions; five in 2/7 repetitions
						Non-reproducible extra-bands were always observed
Badger (*Meles meles*)	Fur	25	0	25	2	
Stone marten (*Martes foina)*	Feces	15	0	15	1 + 2	
Weasel (*Mustela nivalis*)	Feces	15	0	15	2	
Fox (*Vulpes vulpes*)	Peripheral blood	14	0	14	1 + 2	
Hare (*Lepus europaeus*)	Peripheral blood	23	0	23	2	
Bovines (various breeds)	Peripheral blood	23	0	23	1 + 2	

*All amplifications have been repeated at least twice, always in the presence of the positive control (BDF laboratory mouse). Sequencing has been performed on amplicons obtained with our new primers. 1: primers by Ref. (31); 2: newly designed primers*.

*?: variable among repetitions*.

## Concluding Remarks

We have presented in this study a complementary and partially alternative interpretation for one of the most debated cases in public health of the past decade. Our bioinformatic searches and screening of human and animal samples pointed out the pivotal role played, in the diagnostic PCR, by the strong homology between the XMRV genome and repetitive sequences, often of retroviral origin, of house mouse’s genome. Furthermore, the XMRV genome results composed by discrete blocks which also occur in the human genome, in association with (or as part of) interspersed repeated elements. This evidence, together with the results of our amplifications, suggest to re-evaluate the role of contaminations of PCR reagents or air-driven amplicons. Contaminants would have acted uniformly for all the samples considered, while our screening yielded species-specific results, with the unique “robust” and correct amplification obtained in the only case of documented genomic integration of a PreXMRV element, as reported by Cingoz et al. (34). This paper searched for XMRV and parental proviruses integrations in 48 laboratory strains and 46 wild-derived mice: XMRV was never detected by these authors in any murine genome. On the contrary, its parental proviruses PreXMRV 1 and 2 were the only elements found to be integrated in about a half of the laboratory strains and an exiguous minority of wild mice (3/46). Once again, these findings state the question of whether this retrovirus occurs in the “real world,” apart from the cell line where it originated by recombination of the two parental elements and subsequent contaminations.

Repeated DNA sequences are known to be significant and highly dynamic components of genomes, where they play several roles, from centromere and telomere assemblage to epigenetic modulation, from assuring rapid genetic variation to contributing not only to adaptive immune responses but also to many human diseases (37). Nonetheless, the amplification of repetitive DNA is highly problematic, generating deletions and artifacts produced for example by incomplete extension products acting as mega-primers (38) that can lead to false conclusions (39). Laddering effects are among the most frequent artifacts produced by the amplification of repetitive sequences. Indeed, they were consistently observed in all our experiments, notwithstanding the use of newly designed primers with reduced aspecific pairings, and can also be perceived in the gels published by Lombardi et al.

Our data suggest that XMRV is not a suitable template for any diagnostic PCR, because of its strong relatedness to a vast number of repeated DNA elements. This, further worsened by a PCR with primers far from being selective and a low-stringency PCR amplification protocol, has reasonably played a major role in the unreliable associations that have been performed. The reported percentage of the healthy donors which were tested positive for XMRV sequences in the original study (3.7%) is consistent with our results for ME/CFS patients (4.3%), but it is far from raising alarm for public welfare.

## Ethics Statement

All human samples analyzed in the current study are part of the Italian CFS bank, led by AM-CFS-onlus. All enrolled patients and controls (individuals geographically co-localized and sex–age matched to CFS subjects) gave written informed consent in according to the Declaration of Helsinki. The blood from all subjects was collected at IRCCS-Policlinico San Matteo Hospital (Pavia) following the approval of the “Comitato Etico della Fondazione IRCCS Policlinico S. Matteo.” DNA samples from laboratory mice and rats were a kind gift of Prof. Silvia Garagna, University of Pavia. DNA samples from bovine blood were a kind gift of Dr. John Williams, Parco Tecnologico Padano, Lodi. All wild animal samples other than feces and fur belong to dead animals collected in the environment by veterinarians of the National Health Service in the context of routine screenings for zoonoses. Feces and fur were collected in the environment for projects on population biology and biogeography of wild animals involving naturalists from a network of Italian universities.

## Author Contributions

The work was conceived by EC and LL. The bioinformatic analyses were carried out by SP and AB. The experimental work was performed by EC and GL. The manuscript was drafted by SP. All the authors discussed, read, contributed to, and approved the final version.

## Conflict of Interest Statement

The authors declare that the research was conducted in the absence of any commercial or financial relationships that could be construed as a potential conflict of interest.

## Footnotes

^1^www.ncbi.nlm.nih.gov/BLAST.

^2^http://www.repeatmasker.org/cgi-bin/ViewRepeat?id=RLTR4_MM-int.

^3^http://repeatmasker.org.

## References

[1] Carruthers BM Jain AK De Meirleir KL Peterson DL Klimas NG Lerner AM Myalgic encephalomyelitis/chronic fatigue syndrome: working case definition, diagnostic and treatment protocols. J Chronic Fatigue Syndr (2003) 11:7–115. 10.1300/J092v11n01_02

[2] Nacul LC Lacerda EM Pheby D Camopin P Molokhia M Fayyaz S Prevalence of myalgic encephalomyelitis/chronic fatigue syndrome (ME/CFS) in three regions of England: a repeated, cross sectional study in primary care. BMC Med (2011) 28:91 10.1186/1741-7015-9-91 PMC3170215 21794183

[3] Jason LA Benton MC Valentine L Johnson A Torres-Harding S. The economic impact of ME/CFS: individual and societal costs. Dyn Med (2008) 7:6. 10.1186/1476-5918-7-6 18397528 PMC2324078

[4] Holmes GP Kaplan JE Stewart JA Hunt B Pinsky PF Schonberger LB. A cluster of patients with a chronic mononucleosis-like syndrome. Is Epstein–Barr virus the cause? JAMA (1987) 257:2297–302. 10.1001/jama.257.17.2297 3033337

[5] Levine PH Snow PG Ranum BA Paul C Holmes MJ. Epidemic neuromyasthenia and chronic fatigue syndrome in west Otago, New Zealand. A 10-year follow-up. Arch Intern Med (1997) 157:750–4. 10.1001/archinte.1997.00440280064005 9125006

[6] Strickland PS Levine PH Peterson DL O’Brien K Fears T Neuromyasthenia and chronic fatigue syndrome (CFS) in Northern Nevada/California: a ten-year follow-up of an outbreak. J Chronic Fatigue Syndr (2001) 9:3–14. 10.1300/J092v09n03_02

[7] Underhill RA Myalgic encephalomyelitis/chronic fatigue syndrome: an infectious disease. Med Hypotheses (2015) 85:765–73. 10.1016/j.mehy.2015.10.011 26604026

[8] Lorusso L Mikhaylova SV Capelli E Ferrari D Ngonga GK Ricevuti G Immunological aspects of chronic fatigue syndrome. Autoimmun Rev (2009) 8:287–91. 10.1016/j.autrev.2008.08.003 18801465

[9] Eymard D Lebel F Miller M Turgeon F. Human herpesvirus 6 and chronic fatigue syndrome. Can J Infect Dis (1993) 4:199–202. 10.1155/1993/414602 22346448 PMC3250792

[10] Alter HJ Mikovits JA Switzer WM Ruscetti FW Lo S-C Klimas N A multicenter blinded analysis indicates no association between chronic fatigue syndrome/myalgic encephalomyelitis and either xenotropic murine-leukemia virus-related virus or polytropic murine leukemia virus. Mbio (2012) 3:e266–212. 10.1128/mBio.00266-12 PMC3448165 22991430

[11] Tsai SY Yang TY Chen HJ Chen CS Lin WM Shen WC Increased risk of chronic fatigue syndrome following herpes zoster: a population-based study. Eur J Clin Microbiol Infect Dis (2014) 33:1653–9. 10.1007/s10096-014-2095-x 24715153

[12] Urisman A Molinaro RJ Fischer N Plummer SJ Casey G Klein EA Identification of a novel gamma-retrovirus in prostate tumors of patients homozigous for R462Q RNASEL variant. PLoS Pathog (2006) 2:e25 10.1371/journal.ppat.0020025 16609730 PMC1434790

[13] Lo SC Pripuzova N Li B Komaroff AL Hung GC Wang R Detection of MLV-related virus gene sequences in blood of patients with chronic fatigue syndrome and healthy controls. Proc Natl Acad Sci U S A (2010) 107:15874–9. 10.1073/pnas.1012780107 20798047 PMC2936598

[14] Delviks-Frankenberry KD Cingoz O Coffin JM Pathak VK. Recombinant origin, contamination, and de-discovery of XMRV. Curr Opin Virol (2012) 2:1–9. 10.1016/j.coviro.2012.06.009 22818188 PMC3426297

[15] Singh IR Gorzynski JE Drobysheva D Bassit L Schinazi RF. Raltegravir is a potent inhibitor of XMRV, a virus implicated in prostate cancer and chronic fatigue syndrome. PLoS One (2010) 5:e9948. 10.1371/journal.pone.0009948 20376347 PMC2848589

[16] Robinson MJ Erlwein O McClure MO Xenotropic murine leukemia virus-related virus (XMRV) does not cause chronic fatigue. Trends Microbiol (2011) 19:525–9. 10.1016/j.tim.2011.08.005 21978843

[17] Simmons G Glynn SA Komaroff AL Mikovits JA Tobler RH Hackett J Failure to confirm XMRV/MLVs in the blood of patients with chronic fatigue syndrome: a multi-laboratory study. Science (2011) 334:814–7. 10.1126/science.1213841 21940862 PMC3299483

[18] van Kuppeveld FJM de Jong AS Lankem KH Verhaegh GW Melchers WJG Swanink CMA Prevalence of xenotropic murine leukaemia virus-related virus in patients with chronic fatigue syndrome in the Netherlands: retrospective analysis of samples from an established cohort. BMJ (2010) 340:c1018. 10.1136/bmj.c1018 20185493 PMC2829122

[19] Mendoza R Silverman RH Klein EA Miller AD. No biological evidence of XMRV in blood or prostatic fluid from prostate cancer patients. PLoS One (2012) 7:e36073. 10.1371/journal.pone.0036073 22615749 PMC3353987

[20] Johnson AD Cohn CS Xenotropic murine virus-related virus (XMRV) and the safety of blood supply. Clin Microbiol Rev (2016) 29:749–57. 10.1128/CMR.00086-15 27358491 PMC5010753

[21] Das Gupta J Luk KC Tang N Gaughan C Klein EA Kandel ES Absence of XMRV and closely related viruses in primary prostate cancer tissues used to derive the XMRV-infected cell line 22Rv1. PLoS One (2012) 7:e36072. 10.1371/journal.pone.0036072 22615748 PMC3353988

[22] Hue S Gray ER Gall A Katzourakis A Tan CP Houldcroft CJ Disease-associated XMRV sequences are consistent with laboratory contamination. Retrovirology (2010) 7:111. 10.1186/1742-4690-7-111 21171979 PMC3018392

[23] Knox K Carrigan D Simmons G Teque F Zhou Y Hackett J Jr No evidence of murine-like gammaretroviruses in CFS patients previously identified as XMRV infected. Science (2011) 333:94–7. 10.1126/science.1204963 21628393

[24] Alberts B. Retraction. Science (2011) 334:1636. 10.1126/science.334.6063.1636-a 22194552

[25] Urisman A Molinaro RJ Fischer N Plummer SJ Casey G Klein EA Retraction: identification of a novel gamma-retrovirus in prostate tumors of patients homozigous for R462Q RNASEL variant. PLoS Pathog (2012) 8:10.1371 10.1371/annotation/7e2efc01-2e9b-4e9b-aef0-87ab0e4e4732 PMC3445601 23028303

[26] Lo SC Pripuzova N Li B Komaroff AL Hung GC Wang R Detection of MLV-related virus gene sequences in blood of patients with chronic fatigue syndrome and healthy blood donors. Proc Natl Acad Sci U S A (2012) 109:346 10.1073/pnas.1119641109 22203980 PMC3252929

[27] Voisset C Weiss RA Griffiths DJ Human RNA “rumor” viruses: the search for novel human retroviruses in chronic disease. Microbiol Mol Biol Rev (2008) 72:157–96. 10.1128/MMBR.00033-07 18322038 PMC2268285

[28] Jurke A Bannert N Brehm K Fingerle V Kempf VAJ Kompf D Serological survey of *Bartonella* spp., *Borrelia burgdorferi, Brucella* spp., *Coxiella burnetii, Francisella tularensis, Leptospira* spp., *Echinococcus*, Hanta-, TBE- and XMR-virus infection in employees of two forestry enterprises in North Rhine–Westphalia, Germany, 2011–2013. Int J Med Microbiol (2015) 305:652–62. 10.1016/j.ijmm.2015.08.015 26422407

[29] Otra E Garcia-Escudero M Mena Duran AV Monsalve V Cerda-Olmedom G Lack of evidence for retroviral infections formerly related to chronic fatigue in Spanish fibromialgia patients. Virol J (2013) 10:332–42. 10.1186/1743-422X-10-332 24216038 PMC4226024

[30] Irlebeck DM Vernon SD McCleary KK Bateman L Klimas NG Lapp CW No association found between the detection of either murine leukemia virus-related virus or polytrophic murine leukemia virus and chronic fatigue syndrome in a blinded, multi-site, prospective study by the establishment and use of the SolveCFS BioBank. BMC Res Notes (2014) 7:461–71. 10.1186/1756-0500-7-461 25092471 PMC4236736

[31] Lombardi VC Ruscetti FW Das Gupta J Pfost MA Hagen KS Peterson DL Detection of an infectious retrovirus, XMRV, in blood cells of patients with chronic fatigue syndrome. Science (2009) 326:585–9. 10.1126/science.1179052 19815723

[32] Blais J Lavoie SB Giroux S Bussières J Lindsay C Dionne J Risk of misdiagnosis due to allele dropout and false-positive PCR artifacts in molecular diagnostics – analysis of 30,769 genotypes. J Mol Diagn (2015) 17:505–14. 10.1016/j.jmoldx.2015.04.004 26146130

[33] Fukuda K Straus SE Hickie I Sharpe MC Dobbins JG Komaroff A. The chronic fatigue syndrome: a comprehensive approach to its definition and study. International chronic fatigue syndrome study group. Ann Intern Med (1994) 121:953–9. 10.7326/0003-4819-121-12-199412150-00009 7978722

[34] Cingoz O Paprotka T Delviks-Frankenberry KA Wildt S Hu W-S Pathak VK Characterization, mapping, and distribution of the two XMRV parental proviruses. J Virol (2012) 86:328–38. 10.1128/JVI.06022-11 22031947 PMC3255884

[35] Patterson D Costa A Down syndrome and genetics – a case of linked histories. Nat Rev Genet (2005) 6:137–47. 10.1038/nrg1525 15640809

[36] Reverse F Garcia JA. Molecular biology of potyviruses. Adv Virus Res (2015) 92:101–99. 10.1016/bs.aivir.2014.11.006 25701887

[37] Hartl DL. Molecular melodies in high and low C. Nat Rev Genet (2000) 1:145–9. 10.1038/35038580 11253654

[38] Odelberg SJ Weiss RB Hata A White R. Template-switching during DNA synthesis by *Thermus aquaticus* DNA polymerase I. Nucleic Acids Res (1995) 23:2049–57. 10.1093/nar/23.11.2049 7596836 PMC306983

[39] Hommelsheim CM Frantzeskakis L Huang M Ulker B. PCR amplification of repetitive DNA: a limitation to genome editing technologies and many other applications. Sci Rep (2014) 4:5052–65. 10.1038/srep05052 24852006 PMC4031481

